# Molecular characterization of *Staphylococcus aureus* isolates from various healthcare institutions in Nairobi, Kenya: a cross sectional study

**DOI:** 10.1186/s12941-016-0171-z

**Published:** 2016-09-20

**Authors:** Geoffrey Omuse, Kristien Nel Van Zyl, Kim Hoek, Shima Abdulgader, Samuel Kariuki, Andrew Whitelaw, Gunturu Revathi

**Affiliations:** 1 Department of Pathology, Aga Khan University Hospital Nairobi, P.O. Box 30270-00100, Nairobi, Kenya; 2 Division of Medical Microbiology, Department of Pathology, Stellenbosch University, P. O. Box 19063, Western Cape, South Africa; 3 Division of Medical Microbiology, Department of Pathology, Faculty of Health Sciences, University of Cape Town, South Africa, P.O. Box 7925, Cape Town, South Africa; 4 Center of Microbiology Research, Kenya Medical Research Institute, P.O. Box 54840-00200, Nairobi, Kenya

**Keywords:** *Staphylococcus aureus*, MRSA, MSSA, Kenya

## Abstract

**Background:**

*Staphylococcus aureus* (*S. aureus*) has established itself over the years as a major cause of morbidity and mortality both within the community and in healthcare settings. Methicillin resistant *S. aureus* (MRSA) in particular has been a major cause of nosocomial infections resulting in significant increase in healthcare costs. In Africa, the MRSA prevalence has been shown to vary across different countries. In order to better understand the epidemiology of MRSA in a setting, it is important to define its population structure using molecular tools as different clones have been found to predominate in certain geographical locations.

**Methods:**

We carried out PFGE, MLST, SCC*mec* and *spa* typing of selected *S. aureus* isolates from a private and public referral hospital in Nairobi, Kenya.

**Results:**

A total of 93 *S. aureus* isolates were grouped into 19 PFGE clonal complexes (A–S) and 12 singletons. From these, 55 (32 MRSA and 23 MSSA) representative isolates from each PFGE clonal complex and all singletons were *spa* typed. There were 18 different MRSA *spa* types and 22 MSSA *spa* types. The predominant MRSA *spa* type was t037 comprising 40.6 % (13/32) of all MRSA. In contrast, the MSSA were quite heterogeneous, only 2 out of 23 MSSA shared the same *spa* type. Two new MRSA *spa* types (t13149 and t13150) and 3 new MSSA *spa* types (t13182, t13193 and t13194) were identified. The predominant clonal complex was CC 5 which included multi-locus sequence types 1, 8 and 241.

**Conclusion:**

In contrast to previous studies published from Kenya, there’s marked genetic diversity amongst clinical MRSA isolates in Nairobi including the presence of well-known epidemic MRSA clones. Given that these clones are resident within our referral hospitals, adherence to strict infection control measures needs to be ensured to reduce morbidity and mortality associated with hospital acquired MRSA infections.

## Background

*Staphylococcus aureus* (*S. aureus*) has established itself over the years as a major cause of morbidity and mortality globally both within the community and in healthcare settings [1–3]. Its ability to cause disease is aided not only by its impressive repertoire of virulence factors but also its ability to develop resistance to antibiotics used in its treatment epitomized by the emergence of methicillin resistant *S. aureus* (MRSA). Methicillin resistance is conferred by the *mecA* gene that is carried on a staphylococcal cassette chromosome *mec* (SCC*mec*) and codes for a modified penicillin binding protein (PBP2a). This binding protein has reduced affinity to all beta-lactam and beta-lactam/beta-lactamase inhibitor combination antibiotics [4, 5]. In Africa, the MRSA prevalence has been shown to vary across different countries with a prevalence as low as 7 % reported in Madagascar and as high as 82 % in Egypt [6]. This marked variation could be due to different environmental determinants or simply due to a difference in the genetic diversity of *S. aureus*. In Kenya, there is a marked difference in reported MRSA prevalence in clinical isolates within Nairobi with one recent study reporting a prevalence of 3.7 % while another reported 87.2 % [7, 8]. In order to better understand the epidemiology of MRSA, it is important to define its population structure. Molecular characterization helps in identifying clonal populations which can help in surveillance and investigation of outbreaks.

There is a growing interest in the characterization of MRSA isolates and this stems primarily from its role as a major cause of hospital and community acquired infections [1, 9, 10]. There are various molecular methods used, the more common ones include multi-locus sequence typing (MLST), pulse field gel electrophoresis (PFGE), staphylococcal protein A (*spa*) typing and SCC*mec* typing [11]. Despite *S. aureus* having a very diverse clonal population, MLST studies have shown that a small set of clonal complexes (CC) are associated with most of the MRSA epidemics. These include CC5, CC22, CC30, CC45 and CC80 [6, 12, 13]. A clonal complex can have several sequence types, however the multi-locus sequence types that are regarded as the founders in these clonal complexes are ST5, ST22, ST30, ST45 and ST80 respectively [14]. As regards *spa* types, it has been shown that particular ones are more predominant in certain regions. For example t030 is quite predominant in hospitals in Turkey [15], t042 and t044 are more common in North Africa while t008 is common in the US [16]. Unfortunately, the molecular epidemiology of MRSA in Africa is not very well described. Most of the studies carried out in Africa characterizing MRSA have emanated from a few countries namely Tunisia, Nigeria, South Africa, Algeria and Egypt [6]. There are very few studies from East Africa that have reported on the molecular characterization of *S. aureus* presumably due to lack of readily available technical expertise and laboratory facilities. A study done in Kenya looking at carriage of *S. aureus* by inpatients in a government hospital found that only 6 out of 86 (7 %) *S. aureus* isolates were MRSA and they all belonged to the same clone (MLST ST239; *spa* type t037) [17]. This clone is a globally distributed hybrid of ST8 and ST30 and is known to be responsible for several outbreaks in different continents [18–21]. The only other study from Kenya did not report on *spa* or multi locus sequence types [8].

We set out to characterize selected *S. aureus* isolates from different hospitals in Nairobi, Kenya in order to identify which clonal lineages are present and further shed light on the molecular epidemiology of both MSSA and MRSA in Kenya.

### Methods

We obtained archived methicillin susceptible (MSSA) and MRSA isolates from 2 hospitals in Nairobi, Kenya collected between January 2010 and July 2013. The hospitals included a government hospital whose samples we obtained through the Kenya Medical Research Institute (KEMRI) and the Aga Khan University Hospital Nairobi (AKUHN) which is a private referral hospital with a network of satellite clinics and laboratories spread in and around Nairobi as well as different parts of the country. The isolates from the government hospital were part of a previous study done to determine prevalence of MRSA carriage in a paediatric ward and the rest of the isolates were from clinical specimens submitted to the AKUHN laboratory for routine culture and sensitivity. These were convenience isolates that were not collected through a well-structured, formal and documented process. All isolates were stored at −80 °C and grown overnight on sheep blood agar plates at 37 °C.

### *S. aureus* identification

All isolates were confirmed to be *S. aureus* using routine bench identification methods which included growth characteristics on sheep blood agar, gram stain, catalase, coagulase, deoxyribonuclease (DNase) and mannitol fermentation tests. A cefoxitin screen using a 30 µg disc (Oxoid, United Kingdom) was performed to distinguish MSSA from MRSA. Isolates with a diameter ≤21 mm were classified as MRSA.

### Antibiotic susceptibility

Antibiotic susceptibility was only available for the MRSA isolates obtained from AKUHN. These were performed on Vitek 2 (version 4.01, bioMerieux, Marcy-l’Etoile, France) an automated bacterial identification system that performs antibiotic susceptibility using broth dilution and interpretation based on Clinical Laboratory Standards Institute (CLSI) antimicrobial susceptibility guidelines [22]. Multidrug resistance (MDR) was defined as resistance to three or more drug classes.

### DNA derivation

Isolates were grown on blood agar plates (National Health Laboratory Services Media Lab, Cape Town, South Africa) at 37 °C overnight. After incubation, 4–5 large colonies were re-suspended in 200 µL nuclease free water. The samples were incubated at 95 °C for 30 min, followed by −80 °C for 30 min and centrifuged for 10 min at 14,000×*g* when thawed. The supernatant containing DNA was carefully aspirated without disturbing the pellet of cell debris and stored as DNA aliquots at −20° C until further use.

### PFGE

PFGE based on *Sma*I macrorestriction analysis was performed using the CDC laboratory protocol for *S. aureus* [23]. The PFGE was run on a CHEF DR III system (Bio-Rad, California, United States of America) with optimum settings as follows: initial 5 s, switch 30 s, run time 29 h, voltage 6 V/cm and a SeaKem Gold agarose (Lonza, Rockland, USA) gel concentration of 1.4 %. *S. aureus* NCTC 8325 was used as a control in each gel run. Gels were visualized an Alliance 2.7 (UVItec, Cambridge, United Kingdom) gel documentation system after staining with 10 mg/mL ethidium bromide. Analysis of PFGE clusters was performed using the BioNumerics software package (Applied Maths, Sint-Martens-Latem, Belgium), using the Dice coefficient, and visualized as a dendrogram by the unweighted-pair group method, using average linkages with 1 % tolerance and 1 % optimization settings. In order to define a cluster, a cutoff of 80 % similarity was used.

### SCC*mec* typing

SCC*mec* typing was performed using multiplex PCR as described by Milheirico et al. [24]. All assays were performed in a GeneAmp 9600 thermocycler (Applied Biosystems). The optimal cycling conditions were the following: 95 °C for 5 min; 35 cycles of 95 °C for 45 s, 57 °C for 45 s, and 72 °C for 1 min; and a final extension at 72 °C for 10 min. Each PCR mixture contained 0.5 µL of the primers listed in Table 1, KAPA2G Robust HotStart ReadyMix PCR (KAPA biosystems) which contains KAPA2G Robust HotStart DNA Polymerase (1 U/25 µL reaction) in a proprietary reaction buffer containing dNTPs (0.2 mM of each dNTP at 1X), MgCl_2_ (2 mM at 1X), 0.3 µL (3 mM) additional MgCl_2_, 10.7 µL of PCR grade water and genomic DNA in a final volume of 25 µL. The following *S. aureus* isolates were used as controls: BAA-38, BAA-1681, BAA-39, BAA-1680, BAA-1688 and BAA-42 for SCC*mec* types I–VI respectively. The PCR products were resolved in a 1 % SeaKem Gold Agarose (Lonza, Rockland, USA) gel in 0.5 % Tris–borate-ethylene-diamine-tetra-acetic acid (EDTA) buffer (Bio-Rad, Hercules, CA) at 4 V/cm for 2.5 h and were visualized with ethidium bromide.

**Table 1:** Primers used in the updated version of SCC*mec* multiplex PCR

Primer name	Primer sequence (5 33)	Primer specificity (SCC*mec* type, region)	Amplicon size (bp)	Conc. (µM)
CIF2 F2	TTCGAGTTGCTGATGAAGAAGG	I, J1 region	495	0.4
CIF2 R2	ATTTACCACAAGGACTACCAGC			0.4
ccrC F2	GTACTCGTTACAATGTTTGG	V, *ccr* complex	449	0.8
ccrC R2	ATAATGGCTTCATGCTTACC			0.8
RIF5 F10	TTCTTAAGTACACGCTGAATCG	III, J3 region	414	0.4
RIF5 R13	ATGGAGATGAATTACAAGGG			0.4
SCCmec V J1 F	TTCTCCATTCTTGTTCATCC	V, J1 region	377	0.4
SCCmec V J1 R	AGAGACTACTGACTTAAGTGG			0.4
dcs F2	CATCCTATGATAGCTTGGTC	I, II, IV, and VI, J3 region	342	0.8
dcs R1	CTAAATCATAGCCATGACCG			0.8
ccrB2 F2	AGTTTCTCAGAATTCGAACG	II and IV, *ccr* complex	311	0.8
ccrB2 R2	CCGATATAGAAWGGGTTAGC			0.8
kdp F1	AATCATCTGCCATTGGTGATGC	II, J1 region	284	0.2
kdp R1	CGAATGAAGTGAAAGAAAGTGG			0.2
SCCmec III J1 F	CATTTGTGAAACACAGTACG	III, J1 region	243	0.4
SCCmec III J1 R	GTTATTGAGACTCCTAAAGC			0.4
mecI P2	ATCAAGACTTGCATTCAGGC	II and III, *mec* complex	209	0.8
mecI P3	GCGGTTTCAATTCACTTGTC			0.8
mecA P4	TCCAGATTACAACTTCACCAGG	Internal positive control	162	0.8
mecA P7	CCACTTCATATCTTGTAACG			0.8

### *spa* typing

This was done using the following primers: 1095 F: 5′-AGACGATCCTTCGGTGAGC-3′ and 1517R: 5′-GCTTTTGCAATGTCATTTACTG-3′. PCR reactions consisted of 12.5 uL of KAPA2G Robust HotStart ReadyMix PCR (KAPA biosystems) which contains KAPA2G Robust HotStart DNA Polymerase (1 U per 25 µL reaction) in a proprietary reaction buffer containing dNTPs (0.2 mM of each dNTP at 1X), MgCl2 (2 mM at 1X), 0.5 μM of primers and genomic DNA in a final volume of 25 µL. PCR conditions were 95 °C for 6 min; 30 cycles each of 95 °C for 45 s, 64 °C for 45 s, and 72 °C for 60 s; and a final extension at 72 °C for 6 min. Sequencing was outsourced to inqaba biotec, a biotechnology company based in Pretoria, South Africa. Using the Ridom *spa* server (http://www.spa.server.ridom.de), *spa* sequences were automatically assigned to *spa* types. Sequence types and clonal complexes (*sp*a-CC) were assigned where possible using Based Upon Repeat Patterns (BURP) grouping analysis from the Ridom StaphType software (version 1.4; Ridom GmbH, Würzburg, Germany). For BURP analysis, default parameters were used which allows *spa* types with maximum 4 genetic differences to be grouped into one cluster resulting in a calculated cost between members of a group being less than or equal to 4.

### MLST

MLST was done on representative isolates from each PFGE clonal complex and selected singletons according to the protocol published by Enright et al. [25]. The PCRs were carried out as uniplex reactions consisting of 1 µM of the forward and reverse primers, 12.5 µL of 2× KAPA Taq ReadyMix (KAPA Biosystems), 2.5 mM MgCl_2_, 1 µL of template DNA and nuclease free water up to 25 µL. The PCR conditions were 95 °C for 5 min, followed by 30 cycles of 95 °C for 45 s, 56 °C for 45 s and 72 °C for 1 min. A final elongation step was carried out at 72 °C for 10 min. 5 µL of the PCR product was visualised with gel electrophoresis at 120 V for 1 h. Sequencing was performed on the remainder of the PCR product by Inqaba Biotechnical Industries (Pty) Ltd (Pretoria, South Africa). Sequences were inspected and trimmed in BioEdit Sequence Alignment Editor using reference sequences for each of the seven loci. A consensus sequence was generated from the forward and reverse sequences and used to generate sequence types (STs) on the *S. aureus* MLST database (http://www.saureus.beta.mlst.net/#). Isolates that were not typed by MLST were assigned STs using BURP analysis. Isolates with the same PFGE clonal complex and *spa* type were assigned the same STs. MLST clonal complexes (MLST-CC) were determined using a Java applet found at http://www.eburst.mlst.net that uses the eBURST algorithm. The default setting was used in which STs that share identical alleles at 6 or 7 of MLST loci are put in the same group. Where there was a discrepancy between the CC determined using eBURST and BURP, we considered the MLST-CC as the correct one.

## Results

**Fig. 1:**
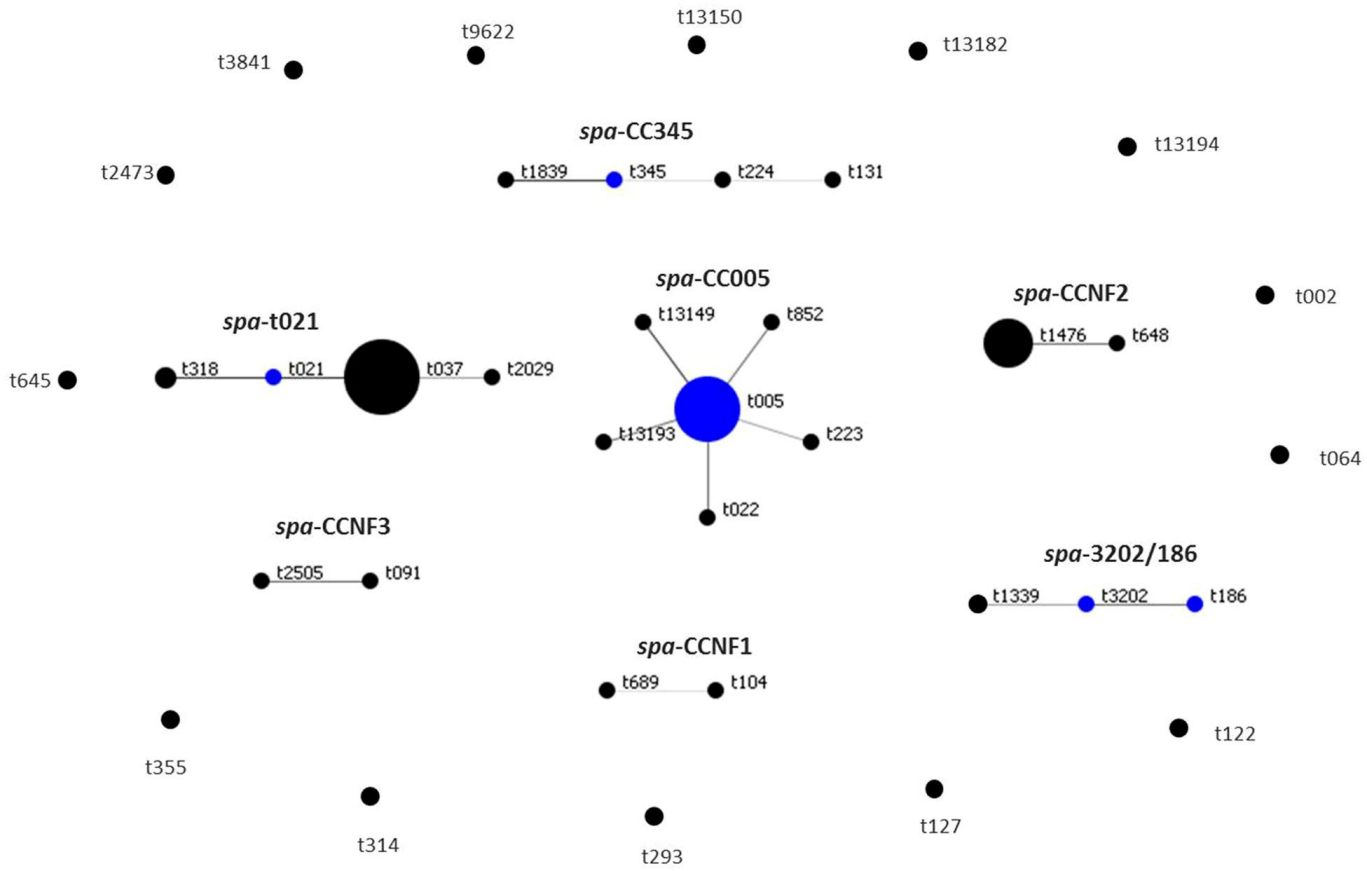
Based upon repeat pattern clustering analysis for all identified *S. aureus* isolates: the clustering analysis resulted in seven *spa*-clonal complexes and 14 singletons. The *blue circle* represents the group founder and the circle size is proportional to the frequency of the *spa* type

A total of 93 *S. aureus* isolates underwent PFGE. These were subsequently grouped into 19 PFGE clonal complexes (A–S) and 12 singletons. From these, 55 (32 MRSA and 23 MSSA) representative isolates from each PFGE clonal complex and all singletons were *spa* typed. This comprised 41 isolates from AKUHN and 14 from KEMRI. In total, there were 18 different MRSA *spa* types and 22 different *spa* types amongst the MSSA. The predominant MRSA *spa* type was t037 comprising 40.6 % (13/32) of all MRSA. In contrast, the MSSA were quite heterogeneous, only 2 out of 23 MSSA shared the same *spa* type. Two new MRSA *spa* types (t13149 and t13150) and 3 new MSSA *spa* types (t13182, t13193, t13194) were identified as shown in Table 2. Three *spa* types (t005, t318 and t1476) were found in both MSSA and MRSA. BURP analysis for both MSSA and MRSA revealed 7 spa-clonal complexes and 14 singletons as shown in Fig. 1. The predominant *spa*-CC was *spa*-CC005 which included the new MRSA *spa* type 13149. SCC*mec* type-III [3A] was the predominant type followed by SCC*mec*-IV [2B]. Only one MRSA isolate was non-typeable using the SCC*mec* protocol published by Milheirico et al. [24]

**Table 2:** Molecular characterization of methicillin susceptible and resistant *Staphylococcus aureus*

Isolate No.	Hospital	Sample	ID	*spa* type	*spa*-CC	SCC*mec* type	MLST/*spa* ST	MLST CC	PFGE CC	PFGE pulsotype
36	AKUHN	Pus swab	MSSA	t645	sng		1841^a^	121	A	A2
84	AKUHN	Pus swab	MSSA	t314	sng		121^a^	121	B	B2
78	AKUHN	Pus swab	MSSA	t355	sng		152^a^	152	C	C1
48	AKUHN	Pus swab	MSSA	t355	sng		152^b^	152	C	C4
91	AKUHN	Nasal swab	MRSA	t005	5	IV	22^a^	22	D	D2
28	AKUHN	Nasal swab	MRSA	t005	5	IV	22^b^	22	D	D3
83	AKUHN	Sputum	MRSA	t005	5	IV	22^b^	22	D	D3
89	AKUHN	Pus swab	MRSA	t13149^d^	5	IV	ND^c^		D	D1
22	KEMRI	Nasal swab	MRSA	t022	5	IV	22^a^	22	E	E2
75	AKUHN	Blood	MRSA	t9622	sng	IV	ND^c^		E	E1
15	AKUHN	Pus swab	MSSA	t005	5		22^a^	22	F	F6
12	AKUHN	Pus swab	MSSA	t223	5		22^a^	22	G	G2
71	AKUHN	Pus swab	MSSA	t122	sng		30^a^	30	H	H1
88	AKUHN	Tracheal aspirate	MSSA	t318	sng		30^a^	30	I	I5
16	AKUHN	Pus swab	MSSA	t021	21		30^a^	30	I	I7
23	AKUHN	Sputum	MRSA	t1339	3202/186	UT	88^a^	88	J	J2
49	KEMRI	Nasal swab	MRSA	t3202	3202/186	UT	ND^c^		J	J1
19	AKUHN	Axillary swab	MSSA	t3841	sng		672^a^	672	K	K2
6	AKUHN	Nasal swab	MRSA	t091	NF3	V	789^a^	7	L	L2
69	AKUHN	Pus swab	MSSA	t2505	NF3		789^a^	7	L	L1
14	AKUHN	Tracheal aspirate	MSSA	t002	sng		5^a^	5	M	M3
79	AKUHN	Pus swab	MRSA	t13150^d^	sng	II	5^a^	5	M	M2
52	AKUHN	Blood	MSSA	t2473	sng		72^a^	72	N	N3
92	AKUHN	Blood	MSSA	t13193^d^	5		22^a^	22	O	O1
87	AKUHN	Blood	MSSA	t127	sng		1^a^	5	P	P3
81	AKUHN	Ear swab	MRSA	t1476	NF2	V	8^a^	5	Q	Q1
45	AKUHN	Pus swab	MSSA	t1476	NF2		8^a^	5	Q	Q2
31	AKUHN	Pus swab	MSSA	t064	sng		8^a^	5	R	R4
33	KEMRI	Nasal swab	MRSA	t104	NF1	IV	8^a^	5	R	R2
7	KEMRI	Nasal swab	MRSA	t689	NF1	I	ND^c^		R	R1
25	AKUHN	Blood	MRSA	t852	5	IV	ND^c^		R	R5
4	AKUHN	Sputum	MRSA	t037	NF4	III	241^a^	5	S	S1
2	KEMRI	Nasal swab	MRSA	t037	NF4	III	241^b^	5	S	S10
3	KEMRI	Nasal swab	MRSA	t037	NF4	III	241^b^	5	S	S10
1	AKUHN	Blood	MRSA	t037	NF4	III	241^b^	5	S	S11
47	KEMRI	Nasal swab	MRSA	t037	NF4	III	241^b^	5	S	S2
38	KEMRI	Nasal swab	MRSA	t037	NF4	III	241^b^	5	S	S3
34	KEMRI	Nasal swab	MRSA	t037	NF4	III	241^b^	5	S	S4
20	KEMRI	Nasal swab	MRSA	t037	NF4	III	241^b^	5	S	S5
18	KEMRI	Nasal swab	MRSA	t037	NF4	III	241^b^	5	S	S6
37	KEMRI	Nasal swab	MRSA	t037	NF4	III	241^b^	5	S	S7
27	KEMRI	Nasal swab	MRSA	t037	NF4	III	241^b^	5	S	S9
29	AKUHN	Pus swab	MRSA	t2029	NF4	IV	241^a^	5	S	S8
13	AKUHN	Pus swab	MRSA	t037	NF4	III	239/240/241^c^			Sng12
11	AKUHN	Pus swab	MRSA	t037	NF4	III	239/240/241^c^			Sng6
17	AKUHN	Pus swab	MSSA	t13182^d^	sng		ND^c^			Sng 3
44	AKUHN	Urine	MSSA	t13194^d^	sng		ND^c^			Sng7
73	AKUHN	Pus swab	MSSA	t1839	345		ND^c^			Sng5
58	AKUHN	Blood	MSSA	t186	3202/186		88^c^			Sng4
70	AKUHN	Blood	MSSA	t224	345		97^c^			Sng8
21	AKUHN	Pus swab	MRSA	t293	sng	IV	ND^c^			Sng 2
43	KEMRI	NASAL SWAB	MRSA	t318	sng	IV	30^c^			Sng 1
35	AKUHN	Pus swab	MRSA	t345	345	V	ND^c^			Sng10
40	AKUHN	Urine	MRSA	t648	NF2	IV	ND^c^			Sng11
85	AKUHN	Vulval swab	MSSA	t131	345		ND^c^			Sng9

*sng* singleton, *NF* no founder, *UT* untypeable, *ND* not defined on the Ridom database Accessed on 07/10/2015

^a^MLST ST

^b^MLST ST extrapolated based on similar *spa* type and pulsotype

^c^ *spa* ST

^d^New *spa* type

MLST STs were determined and extrapolated for 31 isolates. A total of seven different MRSA and MSSA MLST-CC were identified with the predominant one being MLST-CC 5. This clonal complex comprised STs 1, 5, 8 and 241 as shown in Table 2. An isolate belonging to ST241 (t2029) that was detected in a pus sample from AKUHN hospital was found to harbor SCC*mec* type IV [2B].

Out of the 16 MRSA from AKUHN, 13 were MDR including the two new *spa* types. Resistance was commonly seen to clindamycin, erythromycin and trimethoprim/sulfamethoxazole (TMP/SMX). A number of isolates had intermediate resistance to levofloxacin. However, two isolates were only resistant to beta lactams but susceptible to all other antibiotics including TMP/SMX as shown in Table 3. None of the MRSA was resistant to vancomycin, linezolid, mupirocin, teicoplanin or tigecycline.

**Table 3:** Molecular characterization and antibiotic susceptibility of MRSA isolates from AKUHN

Isolate No.	Sample	*spa* type	SCC*mec* type	Antibiogram		
				R	S	I
21	Pus swab	t293	IV [2B]	TOB, CLI, ERY, LEV	LNZ, TEI, VAN, TET, TIG, MUP, RIF, SXT, MOX	
23	Sputum	t1339	UT	SXT	TOB, MOX, ERY, CLI, VAN, TET, TIG, MUP, RIF, LEV	
79	Pus swab	t13150^a^	II [2A]	ERY, CLI, LEV, MOX, TOB	TET, SXT, VAN, MUP, RIF, TIG, TEI, LNZ	
40	Urine	t648	IV [2B]	–	ERY, CLI, TET, SXT, VAN, MUP, MOX, RIF, TIG, TOB, TEI, LNZ, LEV	
25	Blood	t852	IV [2B]	CLI^#^, ERY	LNZ, MUP, MOX, RIF, SXT, TET, TEI, TIG, TOB, VAN	LEV
35	Pus swab	t345	V [5C]	RIF, SXT, TET, TOB	CLI, ERY, LNZ, MUP, MOX, TEI, TIG, VAN	LEV
13	Pus swab	t037	III [3A]	CLI, ERY, SXT, TOB	LNZ, MUP, MOX, RIF, TET, TEI, TIG, VAN	LEV
11	Pus swab	t037	III [3A]	CLI^#^, SXT, TET, TOB	ERY, LNZ, MUP, MOX, RIF, TEI, TIG, VAN	LEV
89	Pus swab	t13149^a^	IV [2B]	TOB, SXT, ERY	CLI, LNZ, TEI, VAN, TET, TIG, MUP, RIF, LEV, MOX	
75	Blood	t9622	IV [2B]	–	ERY, CLI, TET, SXT, VAN, MUP, MOX, RIF, TIG, TOB, TEI, LNZ	LEV
83	Sputum	t005	IV [2B]	TOB, ERY, CLI^#^	MOX, LNZ, TEI, VAN, TET, TIG, MUP, RIF, SXT	LEV
28	Nasal swab	t005	IV [2B]	ERY, CLI^#^, TOB, LEV	TET, SXT, VAN, LNZ, TEI, MUP, MOX, RIF, TIG	
81	Ear swab	t1476	V [5C]	ERY, CLI^#^, TET, SXT, LEV	VAN, MUP, MOX, RIF, TIG, TEI, LNZ	TOB
6	Nasal swab	t091	V [5C]	SXT, TET, TOB	CLI, ERY, LNZ, MUP, MOX, RIF, TEI, TIG, VAN	LEV
29	Pus swab	t2029	IV [2B]	CLI, ERY, RIF, SXT, TET, TOB	LNZ, MUP, MOX, TEI, TIG, VAN	LEV
1	Blood	t037	III [3A]	ERY, CLI^#^, TET, SXT, RIF, TOB, LEV	VAN, MUP, MOX, TIG, TEI, LNZ	

*R* resistant, S susceptible, *I* intermediate, *ERY* erythromycin, *CLI* clindamycin, *CLI* ^*#*^ inducible clindamycin resistance, *TET* tetracycline, *SXT* trimethoprim/sulfamethoxazole, *VAN* vancomycin, *GENT* gentamicin, *MUP* mupirocin, *MOX* moxifloxacin, *RIF* rifampicin, *TIG* tigecycline, *TOB* tobramycin, *TEI* teicoplanin, *LNZ* linezolid, *LEV* levofloxacin

^a^New spa type

## Discussion

This study reveals a markedly heterogeneous population of *S. aureus* isolates as well as the presence of well described MRSA clonal complexes 5, 22 and 30 that are responsible for several outbreaks worldwide [13, 26]. CC5 has been identified as the major clonal complex causing HA-MRSA in Africa with MRSA ST239/ST241-III [3A] having been identified in several African countries [6]. The main clonal complex in our study was CC5 that included ST 241, a single locus variant of ST 239 also known as the “Brazilian/Hungarian clone”. ST 239 and ST 239 like isolates are well-known epidemic clones responsible for several healthcare associated MRSA outbreaks globally. They have been found to be a cause of hospital acquired infections in other African countries including Algeria, Ghana, Morocco, South Africa and Nigeria [6]. A study done by Aiken et al. [17] in a public hospital that is approximately 40 km from Nairobi identified t037-ST239 as the predominant clone carried by inpatients in a surgical ward. Most of the nasal swabs in our study were obtained from children in a paediatric ward situated in a public referral hospital. The high proportion of t037-ST241 among our MRSA isolates is not necessarily reflective of the true prevalence of this *spa* type in Nairobi due to a selection bias in the manner in which the isolates were collected. Nevertheless, it is quite concerning that a clone known to be associated with MRSA epidemics is resident within hospitals in Nairobi indicating an urgent need for proper infection control interventions and regular surveillance.

Unlike the study by Aiken et al. [17] that only found one MRSA clone, we identified 18 distinct *spa* types amongst the MRSA isolates belonging to very diverse sequence types, including 2 MRSA *spa* types (t13149 and t13150) that have not previously been described. The *spa* type t13150 was found to belong to ST5-II [2A] which has also been found in Nigeria and Senegal [27, 28]. We identified MRSA belonging to ST22 which in Africa has only previously been found in Algeria, Tunisia and South Africa. This clone has been widely associated with hospital epidemics especially in new born units [29]. The “West Australia MRSA-2 clone” (WA-MRSA-2), ST88-IV [2B] which has been reported in Cameroon and Madagascar was not found and the European MRSA clone ST80-IV that has been found in North African countries was not present in our collection. None of the MRSA in this study belonged to *spa* type t008, the prevalent *spa* type associated with the USA300 pulsotype that has been identified as the major cause of community acquired skin and soft tissue infections in North America [30, 31]. Although the isolates included in our study were few, they represent a fairly diverse collection from both a public and private referral hospital and we can therefore conclude that USA300 is not common in Nairobi.

The 23 MSSA belonged to 22 different *spa* types highlighting their marked genetic diversity in contrast to the MRSA. There were 3 *spa* types (t005, t318 and t1476) that were found in both MSSA and MRSA suggesting the possibility of local acquisition of an SCC*mec* element. One of the MSSA *spa* types belongs to t002 which is associated with the MRSA pulsotype USA100 [16]. The *spa* type t064 was also found which is associated with one of the major MRSA clones (ST612- SCC*mec* IV [2B] found in South Africa [32]. ST241 has frequently been associated with SCC*mec* III [3A], however, one isolate belonging to ST241 harbored SCCmec IV [2B] (this SCC*mec* element was more common in AKUHN hospital). This particular clone was previously observed in a large university clinic in Nigeria [33]. SCC*mec* types IV and V are small in size and can be transmitted both in the community and healthcare settings. Potentially, this could result in the emergence of well-known epidemic MRSA clones like the predominant European CA-MRSA clone ST80-IV [2B] whose for bearer is thought to be a PVL-positive MSSA from sub-Saharan Africa that acquired the SCC*mec* IV [2B] [34]. The MSSA strain t021-ST30 has also been associated with a known PVL positive CA-MRSA clone [35].

The multi-drug resistant patterns for the MRSA in this study are in keeping with what has been described in other countries in Africa [17, 32, 36, 37]. Most of the MRSA were resistant to macrolide–lincosamide, tetracycline and sulphonamide group of antibiotics which is fairly common amongst MRSA especially those that are healthcare associated. However two of the isolates showed resistance to only beta lactam antibiotics suggesting that they may be community acquired (based on their molecular structure) given that they belonged to SCC*mec* type IV which has been associated with CA-MRSA.

The major limitation of this study is that the isolates characterized were not collected in a structured and consistent manner and as such the proportions reported do not necessarily represent a true picture of the relative distributions of different clones in Nairobi due to a selection bias. The over representation of nasal swab specimens from a paediatric population from one hospital may have exaggerated the prevalence of t037-ST 241. We also did not carry out MLST and *spa* typing on all isolates due to financial constraints. However, we did ensure that a representative isolate from each PFGE clonal complex was included in the isolates that were further characterized using MLST and *spa* typing.

## Conclusion

To the best of our knowledge, this is the largest study from Kenya that has carried out PFGE, MLST, *spa* and SCC*mec* typing on a diverse collection of MRSA isolates. This study highlights the marked genetic diversity of MSSA and MRSA isolates in Nairobi including the presence of well-known epidemic MRSA clones and new MRSA *spa* types. Given the evolution of *S. aureus* over the years, there is need for continuous surveillance in order to keep track of emerging clones. The existence of epidemic MRSA clones further justifies the need to strengthen infection control measures within our hospitals so as to avoid nosocomial *S. aureus* infections.

## Abbreviations

AKUHN: Aga Khan University Hospital Nairobi
BURP: based upon repeat pattern
CC: clonal complex
CLSI: Clinical Laboratory Standards Institute
DNA: deoxyribonucleic acid
dNTP: deoxynucleotide triphosphate
EDTA: ethylene-diamine-tetraacetic acid
KEMRI: Kenya Medical Research Institute
MDR: multidrug resistance
MLST: multi-locus sequence Type
MSSA: methicillin susceptible *Staphylococcus aureus*
MRSA: methicillin resistant *Staphylococcus aureus*
ND: not defined
NF: no founder
PBP2a: penicillin binding protein 2a
PCR: polymerase chain reaction
*S. aureus*: *Staphylococcus aureus*
SCC*mec*: Staphylococcal cassette chromosome *mec*
ST: sequence type
UT: untypeable
